# Does the Integration of Personalized Ultrasound Change Patient Management in Critical Care Medicine? Observational Trials

**DOI:** 10.1155/2013/946059

**Published:** 2013-12-18

**Authors:** Raoul Breitkreutz, Marco Campo delľ Orto, Christian Hamm, Colleen Cuca, Peter M. Zechner, Tanja Stenger, Felix Walcher, Florian H. Seeger

**Affiliations:** ^1^Emergency Department, Hospital of the City of Frankfurt (Höchst), Gotenstraße 6-8, 65929 Frankfurt am Main, Germany; ^2^Emergency Ultrasound Regional Network and the Frankfurt Institute of Emergency Medicine and Simulation Training, Johann Wolfgang Goethe University Hospital, Marienburgstraße 5-7, 60528 Frankfurt am Main, Germany; ^3^Department of Cardiology, Kerckhoff Klinik Bad Nauheim, Benekestraße 2-8, 61231 Bad Nauheim, Germany; ^4^Deptartment of Anaesthesiology and Intensive Care, Hospital zum Heiligen Geist, Lange Straße, 60311 Frankfurt, Germany; ^5^Landeskrankenhaus Graz West, Abteilung für Innere Medizin, Göttinger Straße 22, 8020 Graz, Austria; ^6^Städtisches Klinikum Neunkirchen, Brunnenstraße 20, 66538 Neunkirchen/Saar, Germany; ^7^Brainfactory, 66288 Bildstock, Germany; ^8^Trauma Surgery, Johann Wolfgang Goethe University Hospital, Theodor Stern Kai 7, 60590 Frankfurt am Main, Germany; ^9^Department of Cardiology, Johann Wolfgang Goethe University Hospital, Theodor Stern Kai 7, 60590 Frankfurt am Main, Germany

## Abstract

*Objective. *To test the influence of personalized ultrasound (PersUS) on patient management in critical care. *Design of the Study*. Prospective, observational, and critical care setting. Four substudies compared PersUS and mobile ultrasound, work distribution, and diagnostic and procedural quality. *Patients and Interventions*. 640 patient ultrasound exams including 548 focused diagnostic exams and 92 interventional procedures. *Main Outcome Measures*. Number of studies, physician's judgement of feasibility, time of usage per patient, and referrals to echo lab. *Results*. Randomized availability of PersUS increased its application in ICU work shifts more than twofold from 33 to 68 exams mainly for detection and therapy of effusions. Diagnostic and procedural quality was rated as excellent/very good in PersUS-guided puncture in 95% of cases. Integrating PersUS within an initial physical examination of 48 randomized cases in an emergency department, PersUS extended the examination time by 100 seconds. Interestingly, PersUS integration into 53 randomized regular ward rounds of 1007 patients significantly reduced average contact time per patient by 103 seconds from 8.9 to 7.2 minutes. Moreover, it lowered the patient referral rate to an echo lab from 20% to 2% within the study population. *Conclusions*. We propose the development of novel ultrasound-based clinical pathways by integration of PersUS.

## 1. Introduction

Point-of-care ultrasound has become more popular in the environment of acute and critical care medicine [1, 2]. Various recommendations on its use mainly address clinical indications related to acute or severe dyspnoea, hypotension and shock, trauma, and abdominal pain [1–4]. The technical concept of a “personal ultrasound imager” and an “ultrasonic stethoscope” is more than 30 years old [5]. There is a strong interest in the integration of ultrasound into clinical pathways and many context-based protocols are available. A novel technology was born when the initial idea of a personalized ultrasound device (PersUS) used like a physician's generic stethoscope was realized [5, 6], a paradigm shift that now supports real point-of-care clinical pathway concepts. In 1978 it was suggested that bringing the echo lab to the patient would be a major step forward [5]. Previously, ultrasound labs contained stationary ultrasound systems, while mobile and hand-carried ultrasound was kept at facilities like emergency departments or intensive care units, making the technology available for multiple users. In fact, “personalized” refers to the size and quick application of the PersUS, like a mobile phone or stethoscope, allowing greater flexibility in its usage. There is an ongoing debate as to whether miniaturization of ultrasound machines can improve patient care. The potential personalized usage opens up the field of ultrasound in acute and critical care medicine for a large group of new users [3]. However, only rarely have concepts or strategies for clinical integrations been tested for the critical care environment and little has been established in the way of clinical integration and workflow [7]. Our aim, therefore, was the analysis of the feasibility of clinical integration, frequency of use, decision making, and time consumption of PersUS implementation in the daily routine.

## 2. Methods

### 2.1. Study Design

Ethical approval was obtained from the Institutional Ethics Committee for Human Studies, University Hospital, Frankfurt am Main, Germany (application number 2/11, 13.1.2011). A prospective observational study with data-controlled acquisition was performed.

### 2.2. Study Setting and Substudies 1–4

Patients were enrolled between July 2010 and September 2011. Personalized ultrasound (PersUS) was performed in four centres of the coauthors and in each case using the Vscan (GE Healthcare, Wauwatosa, WI, USA). After an opening and booting time of 25 seconds, the device allows ultrasound exams with a phased array sector probe (1.7–3.8 Mhz) in B-Mode with harmonic imaging and color-coded flow mapping (color Doppler) on a 3.5-inch screen. Support was provided through the “Vscan gateway” (GE Healthcare, Wauwatosa, WI, USA) for all participating examiners. Physicians were trained in focused ultrasound exams in critical care medicine for general applications of the thorax and abdomen and documented each exam with a notation on the patient chart and/or images or clips for later review within the study protocols. Two investigators were staff attending cardiologists and three investigators were staff attending anaesthesiologists or intensivists. Junior staff with expert knowledge in emergency ultrasound performed substudies 3 and 4. Images or clips were deleted after review by the respective attending or study control centre to ensure anonymity. Alternatively, the study protocol allowed a mobile ultrasound device (MobUS) to be used. By chance this was always a Vivid-i (GE Healthcare, Wauwatosa, WI, USA) with each collaboration partner. To examine a great number of patients with a broad spectrum of diseases, this trial was subdivided into four distinct substudies.

#### 2.2.1. Substudy 1

Our hypothesis was an availability-based increase in the use of PersUS. We tested this hypothesis in two independent intensive care units with two ultrasound-capable physicians (one cardiologist-intensivist and one anesthesiologist-intensivist), respectively. The practitioners had access to the PersUS device—carried on person—on either odd or even days, which was determined randomly.

#### 2.2.2. Substudy 2

Four critical care physicians studied PersUS for planning an execution of ultrasound-guided needle punctures, such as pleural, pericardial, abdominal, or urinary bladder punctures for evacuation of fluid or inserting catheters (pleurocath, pigtail, or suprapubic). Linear analogue self-assessment was used to obtain semiquantitative data of the physician's impression of (i) diagnostic quality and (ii) visual support in ultrasound-guided intervention. Any relative value above 90% was graded as “excellent” and between 80 and 90% as “good” or between 70 and 80% as “satisfactory.”

#### 2.2.3. Substudy 3

We measured the mean duration of a physical examination with PersUS integration according to the decision of a single emergency physician who used PersUS after randomisation by coin toss. Our null hypothesis was that PersUS exam integration does not significantly prolong the examination time. Indications were acute dyspnoea, thorax pain, hypotension, or abdominal pain. The time between examinations begining and end (handshake following exam) was measured by a generic stopwatch. Any influence on patient management due to PersUS was noted. Of note, PersUS was used during the first patient contact and integrated into the examination. All patients needing ultrasound, for example, to exclude pericardial effusion, received the US with the MobUS device as usual.

#### 2.2.4. Substudy 4

In a cardiological and nephrological specialty care unit a PersUS device was randomized between two wards by switching its presence or absence on alternating calendar days. One senior cardiologist alone was informed as the study coordinator of this substudy and allowed to make the PersUS available or remove it from the respective ward. The ward rounds teams, consisting of one or two staff physicians and one attending physician, were blinded to the study aims and differed from day to day. They were instructed to use PersUS whenever it was available and deemed necessary and to note the times and indications of use. Time between beginning and end of ward rounds was measured as well as the number of patients seen and the type and length per ultrasound exam. Most of the examinations during the ward rounds were focused on LV-function as well as effusions. More subtle examinations such as diastolic function of valve regurgitations were sent to the echo lab. The quality of the exam was graded on a Likert scale regarding diagnostic quality as judged by the examiner. All data was then sorted into Group A (control group without PersUS) or Group B (examination with PersUS). Exam time per patient per ward round was calculated. Our null hypothesis was that the exam time per patient would not change significantly by using PersUS on ward rounds.

### 2.3. Study Entry Criteria

Patient inclusion was based on the decision of the participating treating physician. After informed consent, patient-related data and procedure details were logged either on a data acquisition sheet or into a paperless database. If patients were unable to give their informed consent (sedation, dementia) relatives were informed. All interventions were applied only after a generic routine MobUS confirmed the initial findings. Indication was formally only supported by MobUS findings and no additional interventions were performed based on PersUS findings alone. Participating physicians were briefed on the implementation of PersUS. Patients below 18 years of age were excluded from the study. Bias could not be controlled otherwise.

### 2.4. Data Analysis

Physicians interpreted all ultrasound exams at the time of the scan and completed the data acquisition protocols upon exam conclusion. Unless stated otherwise, continuous data is shown as mean ± standard deviation. Box plots show median (bold line), mean (dashed line), 25th and 75th quartiles, whiskers, and all outliers. The Mann-Whitney *U* test was used for descriptive data analysis for comparison between the two groups in substudies 1, 3, and 4. Study size was planned with a fixed number of patients. Power analysis was not applicable for there were no data to precisely predefine our variables. There were no missing data to be excluded.

## 3. Results

### 3.1. Substudy 1: Clinical Integration of PersUS in Intensive Care

Of 31 work shifts (16 without and 15 with PersUS) there were a total of 101 patient exams with 167 ultrasound indications (Table 1). Major indications were diagnostic and related to cardiac anatomy and function as well as pleural effusion (Table 1).

**Figure 1:**
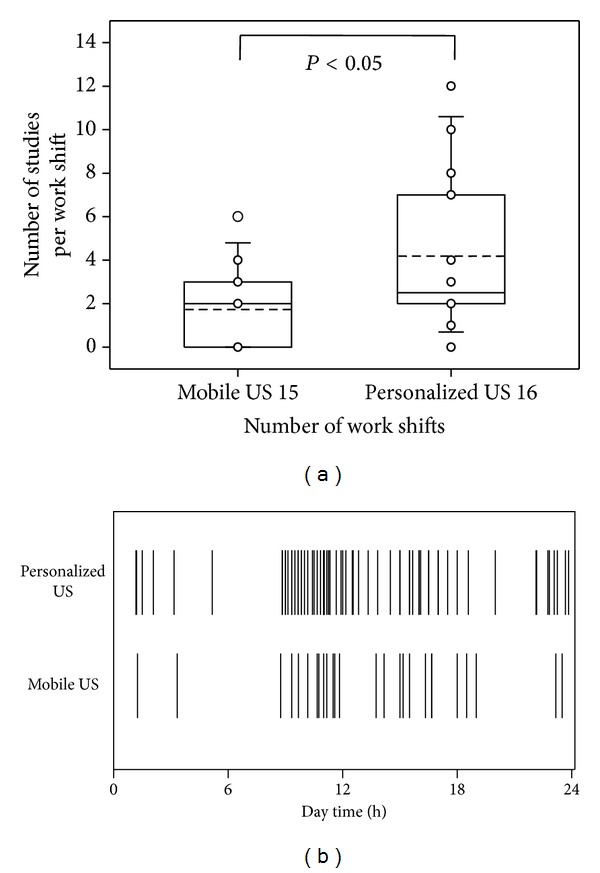
Increased personalized ultrasound use in critical care medicine (substudy 1). Randomized comparison of personalized versus mobile ultrasound. (a) number of exams per work shift; (b) distribution pattern over three work shifts. These results indicate the increase in request of the frequency for ultrasound exams during ward rounds (8 a.m., 1 p.m., 8 p.m.) or shortly thereafter, which can be better implemented with personalized ultrasound.

When only a MobUS was available, 33 patients received a focused ultrasound exam. In contrast, the availability of the PersUS markedly increased the number of patients receiving an ultrasound exam up to 68 (relative change: 106%; Figure 1(a), Table 1) and allowed interventions such as pleural puncture to be applied at an earlier point in time. Mean PersUS operation time per patient was 6.5 ± 3.9 minutes, 1.7 minutes faster than MobUS (Table 1). There was hardly any difference in cardiac evaluations; however, for thoracic and other ultrasound examinations there was a remarkable reduction of hands-on/hands-off time per patient (Table 1). During a 3-shift intensive care system, PersUS was most likely to be requested by the physician directly after the morning ward rounds, followed by afternoon or night shift ward rounds (Figure 1(b)).

### 3.2. Substudy 2: Evaluation of Image Quality and Interventional Support

**Figure 2:**
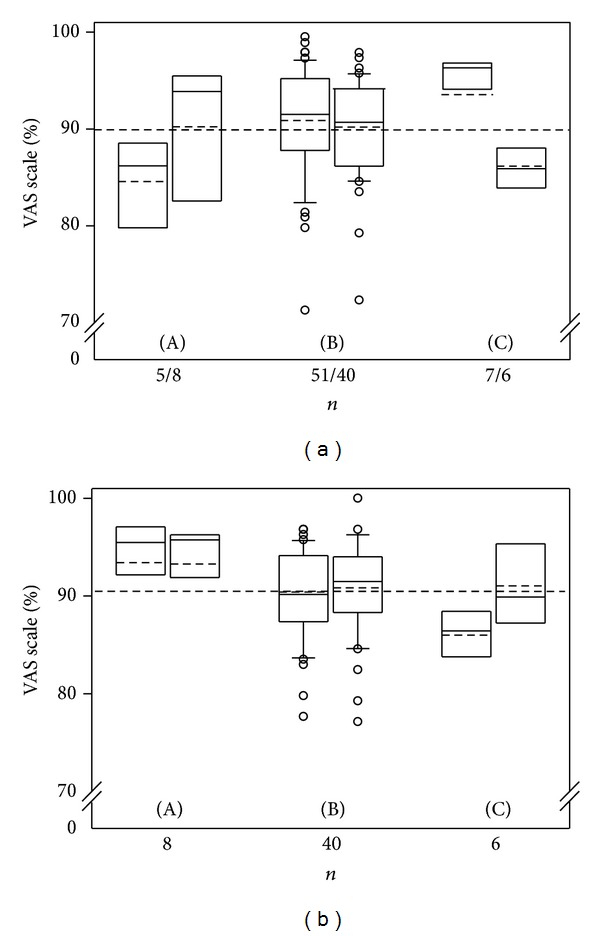
Physician assessment of diagnostic quality, ultrasound-guided interventions, and reproducibility when using a personalized ultrasound device (VAS scale; substudy 2). (A): pericardial effusion; (B): pleural effusion; (C): urinary bladder. (a) Each left boxplot shows diagnostic ultrasound, each right boxplot ultrasound-guided punctures. (b) Physician assessment of feasibility in ultrasound-guided interventions with a personalized device (VAS scale): inclination to use PersUS in future examinations (each left boxplot) and inclination to use PersUS for other anatomical regions (each right boxplot).

Four attending physicians in critical care evaluated diagnostic and image quality of the PersUS exam and its image quality as well as feasibility in interventional procedures (Figure 2(a)). For pleural effusions, which were the most common diagnoses within the study, PersUS was rated “excellent” (Figure 2(a)(B)). PersUS use demonstrating pericardial effusion and urinary bladder status (Figure 2(a)((A), (C))) as well as during PersUS-guided procedural intervention was rated “good,” except in the case of urinary bladder puncture (Figure 2(a)). Numbers for ascites punctures (2) were judged similarly. After PersUS-guided intervention was complete, physician requests for device use in later punctures increased substantially (Figure 2(b)).

### 3.3. Substudy 3: Time Requirements of PersUS

**Figure 3:**
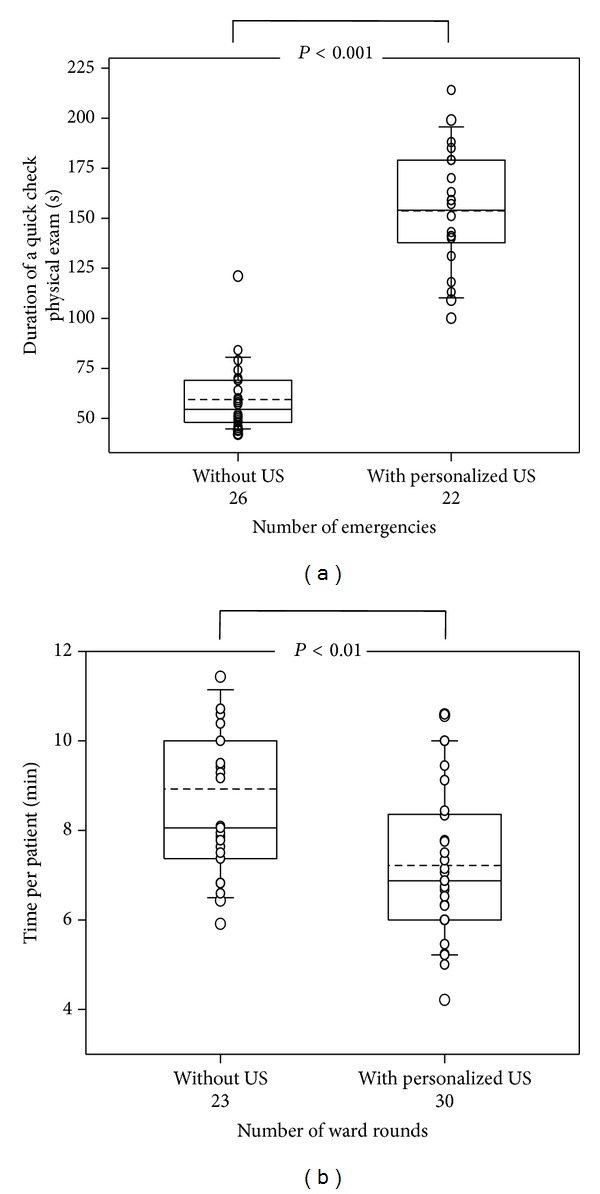
Influence of personalized ultrasound on time when integrated into the physical examination of emergency admissions or within regular ward rounds (substudies 3 and 4). Although mean examination time per emergency patient increased (a), average time consumption per patient on ward rounds markedly decreased (b).

Within nineteen work shifts in an ED, 48 patients with leading symptoms acute dyspnoea (*n* = 21; 44%), abdominal pain (15; 31%), thorax pain (11; 23%), and hypotension/shock (1; 2%) were randomized to be examined on admission either without (Group A; 26 patient admissions, 14 female and 12 male, aged 59 ± 22 years) or with the assistance of PersUS (Group B; 22 patient admissions, 9 female and 13 male, 62 ± 21 years). The duration of this “quick check” initial physical exam in Group A was 59 ± 3 seconds (mean ± SD, 95% CI of mean; 6.8; Figure 3(a)). Integration of PersUS into an initial physical exam directly upon patient admission in the ED allowed the quantification of possible excess time consumption. Although PersUS was integrated easily into this physical exam, it caused a marked prolongation of the examination time to a mean of 154 ± 6 seconds (95% CI 13.2) in Group B (Figure 3(a)). However, in Group B, a change of management in 6/22 (27%) cases as well as valuable additional information for immediate recognition of underlying disease in 19/22 (86%) patients (10 with dyspnoea/thorax pain; 9 with abdominal pain) was registered by the examiner.

### 3.4. Substudy 4: Integration into Critical Care Ward Rounds

PersUS was integrated into 53 regular ward rounds with a total of 1007 patients on two wards. Mean ward round operation time was 142 ± 33 minutes with 18 ± 3 patients per ward.

In 194 of 1007 (19%) patient visits, an ultrasound exam was requested due to one or more indications per patient (Figure 3(b), Table 2). According to the randomization, 133 questions regarding 95 patients in Group A remained unanswered as a result of a strict removal of the PersUS. In Group B, when focused PersUS was available, 134 questions regarding 99 patients could be answered while focused PersUS exams were applied at each respective patient contact. The average PersUS examination time was 3.6 ± 2 minutes per patient and was rated 2.4 ± 0.9 (good to satisfactory). Interestingly, this PersUS integration into the ward rounds management effected a significant reduction of the time needed per patient from a mean of 8.9 minutes to 7.2 minutes (Figure 3(b)). Patient referral to the echo lab for further examination was deemed necessary for 95 of 473 patients (20.1%) in Group A; however, only 12 of 534 patients (2.2%) in Group B with 16 distinct questions were referred. This was mainly the case in request for Doppler examination of diastolic function, which is not yet available in PersUS.

Based on clinical context, ten categories of focused ultrasound examinations were established. (Table 2).

There were no adverse results or effects during any patient exams or interventions with PersUS or MobUS in all substudies.

## 4. Discussion

The main findings of our studies were that personalized ultrasound was safe, feasible, of good quality, and easily implementable into routine critical care work. The availability of personalized ultrasound increased the requests for focused ultrasound exams and offered an image quality comparable to high-quality mobile ultrasound, allowing targeted decision- making. While PersUS extended the examination time in emergency admissions it positively influenced patient management, increased information gain about the underlying disease, reduced the contact times per patient in ward rounds, and lowered the request for patient referral to an echo lab.

### 4.1. Feasibility of Integration into Clinical Operating Processes

Ultrasound and echocardiography in critical care medicine were considered for widespread use [8] and recommended in a recent guideline as a complement to a physical examination in coronary and intensive care units [3]. It has been shown that ultrasound in the ED or ICU supports the early finding of main diagnoses and has the potential to eliminate other differential diagnoses [9, 10]. However, clinical integration concepts are lacking [3, 5]. PersUS allows a more sophisticated integration into the daily workflow and clinical pathways so that ad hoc procedures can be realized.

However, PersUS offers more options: it can complement [11] or replace a complete physical exam while screening [12] or be interwoven with the physical exam or algorithm-like procedures such as the focused assessment of abdominal sonography (FAST). It can be utilised in triage [13, 14] or integrated into the advanced life support as focused echocardiography (FEEL exam) [15–17]. Furthermore, a PersUS could be incorporated into more complex operating procedures such as work shifts and ward rounds.

The ready availability of PersUS increased the number of requests for focused ultrasound examinations. These were not referred to another operator or echo lab but executed as point-of-care exams in real time or shortly thereafter by the same physician determining the indication. The types of indications were related to cardiac chamber dimensions and function as well as pericardial and pleural effusions and reflect recent recommendations for focused echocardiography in cardiology [18]. In critical care and ventilated patients, effusion diagnostic and interventions were the main reasons for the increase of requests, thus reflecting a real need for transcutaneous ultrasound exams in critical care practice.

### 4.2. Is a 3.5-Inch Screen Sufficient to Make Decisions or Guide Punctures?

The PersUS screen size raises concerns about image quality [19], although similar devices have been demonstrated to provide the same accuracy in cardiac sonoanatomy (endo-/pericardial effusion) as high-end echocardiography [20, 21]. We observed a highly reliable image quality for evaluation of effusions and basic cardiac anatomy and function in critical care. Our data suggests that the size itself has little impairment on decision making in real time. In agreement with recent studies of the same type of device [20, 21] we found that this quality was sufficient for real-time punctures of various targets. However, for more advanced examinations, such as diastolic function, MobUS seems to be the better choice.

### 4.3. Time Constraints

One concern of PersUS integration in our study was the investment of up to 10 minutes per ultrasound exam. Time is an essential component in acute care. Early application of ultrasound has been shown to reduce the number of viable diagnoses in the emergency setting [9] and determine outcome [13, 16, 17, 22], leading to calls for documentation of focused ultrasound examination length [18]. For goal-directed echocardiography, mean acquisition time was 10.5 ± 4.2 min [23]. Duration depends on the type of focused exam [3] and the body region (cardiac, lung, abdomen, and multiple regions). Exam times can vary from seconds up to 10 min [23–30]. The screening capability of PersUS allows effusions, for example, to be examined faster in triage [12, 13, 26] or within Advanced Life Support (maximum 10 seconds for a subxiphoidal view) during pauses between chest compressions [25]. In contrast, it was estimated that the comprehensive cardiac or abdominal exams would take more than 20 min [4, 18, 29, 31]. Although we found similar results in our substudies of cardiac diagnostic ultrasound, the integration of a quick-check ultrasound exam such as the one in the acute setting was considerably faster. The PersUS exam in our study was not restricted to cardiac indications [30], contained fewer than 5 questions per patient, and required much less time than was expected [32].

### 4.4. Future Remarks

We suggest combining the physical and PersUS exams into a standard clinical exam protocol [33]. This would yield increased implementation in the clinical context of acute and critical care medicine and cost-effective analysis as calculated for other settings [34–36].

### 4.5. Limitations

Due to the prospective design and the broad number of patients examined in different substudies and hospitals, we did not have the possibility to review all examinations by blinded experts. In addition, blinding of the pictures/movies according to the US device was technically not possible. Therefore, comparison between different US devices might be subjective according to the examiners observation. Moreover, this study does not intend to suggest that focused and personal ultrasound examinations are sufficient to understand the patient's complete physiological state. Complete evaluation of dyspnoea, for example, requires comprehensive echocardiography [18]; however, this is rarely achievable in all critical care units in real time, leaving the treating physician to obtain additional information for a specific clinical problem before the more specialised practitioner is involved. Our study required neither comparison with findings using a standard ultrasound machine and a comprehensive exam nor confirmation of findings from a second expert sonographer. It is not generalisable to other hospitals. However, there is, to our knowledge, no existing gold standard for focused ultrasound combined with clinical examination.

## 5. Conclusions and Key Message

The integration of personalized ultrasound in the daily acute and critical care workflow is safe and easily applied to patient admissions, routine procedures, and ward rounds with little additional time requirement. It will accelerate and improve decision making and interventions. We propose the development of novel ultrasound-based clinical pathways, standard operation procedures, and workflow protocols by integration of PersUS.

## Figures and Tables

**Table 1:** Types of indications for application of a mobile ultrasound device (MobUS) in comparison to a randomized availability of a personalized ultrasound device (PersUS). *N* = 101 patients were examined (33 exams with MobUS, 68 with PersUS) with a total of 167 indications and ultrasound studies.

Category number	Kind of indication	*N* (% of total) MobUS studies	Time (min) Mean ± SD	*N* (% of total) PersUS studies	Time (min) Mean ± SD	Percentage change (%)
1	Cardiac function (visual LVEF, eyeballing)	15 (9)	4.8 ± 1.1	36 (22)	4.4 ± 1.0	114
2	Focused cardiac anatomy and valve assessment	14 (9)	4.7 ± 1.1	27 (17)	4.5 ± 1.0	93
3	Dyspnoea, suspected pleural effusions	7 (4)	14.1 ± 9.9	24 (14)	9.2 ± 4.8	243
4	Pericardial effusion	4 (2)	4.8 ± 2.2	14 (9)	4.4 ± 0.9	250
5	Abdomen/ascites	3 (2)	21.7 ± 7.6	8 (4)	9.9 ± 5.5	166
6	Ultrasound-guided punctures	2 (1)	20 ± 0	5 (3)	12.0 ± 4.5	150
7	Miscellaneous (a)	1 (1)	10 ± 0	2 (1)	4.5 ± 0.7	100
8	Resuscitation	3 (2)	4.0 ± 1.0	1 (1)	4.0 ± 0	n.a.

	Total (167, 100%)	49 (29)	8.2 ± 7.0	118 (71)	6.5 ± 3.9	141

(a) Including groin aneurysm spurium and cubital vein detection for puncture.

n.a.: not applicable. All times as estimated by the examiner from beginning (hands-on) to end (hands-off) of the examination procedure. Descriptive data presentation only. Percentage change was calculated as (the number of PersUS studies minus the number of MobUS studies) divided by the number of MobUS studies.

**Table 2:** Indications for clinical context-based ultrasound requests within routine ward rounds without or with personalized ultrasound (PersUS). Randomized determination of availability of PersUS. Group A did not receive ultrasound within a ward round and indications regularly determined a systematic echocardiography in a laboratory. In contrast, Group B received personalized ultrasound during the ward round.

Category number	Indication for request of a focused exam within ward round	Group A No US	Group B PersUS	Total	Decision for referral to echo lab Group A versus B
No. of patients in ward round		473	534	1007	95 versus 12

No. of patients receiving indications for echocardiography		95	99	194	95 versus 12

1	Focused echo (1)	22	16	38	22/1
2	EF of both ventricles	41	37	78	41/6
3	Pleural effusion (both hemithoraces) including quantification	37	42	83	37/4
4	Ascites, marking for later puncture or puncture (2)	11	8	18	11/1
5	Valve function (3)	3	4	7	3/2
6	Mitral insufficiency (focused assessment prior TOE)	4	0	4	4/0
7	Resuscitation (FEEL protocol)	0	1	1	—
8	Urinary bladder filling state, postrenal failure	1	2	3	1/0
9	Pericardial effusion (exclusion, or size and clinical course)	22	18	40	22/0
10	Pulmonary valve replacement, postinterventional check	2	6	8	2/0
Indications total		133	134	267	133/14

(1) Combined focused TTE including EF, oriented valve morphology and function, left and right ventricular dimensions. Clinical contexts contained focus on hypertension (LV-hypertrophy), right heart pressure overload, NSTEMI (LVEF), atrial fibrillation (valves, LVEF), postintervention (EF, pericardial effusion), pulmonary vein isolation therapy in case of atrial fibrillation (EF, pericardial effusion).

(2) Including 3 cases per group of the request soft tissue or musculoskeletal assessment for hematoma in the groin after coronary angiography or after pacemaker/defibrillator implantation in the anterior chest or shoulder area.

(3) Main issues were focused assessment of aortic valve opening in the elderly.

## Abbreviations

ED: Emergency department
PersUS: Personalized ultrasound
MobUS: Mobile ultrasound.
